# Impairment of Cerebrovascular Hemodynamics in Patients With Severe and Milder Forms of Sickle Cell Disease

**DOI:** 10.3389/fphys.2021.645205

**Published:** 2021-04-20

**Authors:** Liza Afzali-Hashemi, Koen P. A. Baas, Anouk Schrantee, Bram F. Coolen, Matthias J. P. van Osch, Stefan M. Spann, Erfan Nur, John C. Wood, Bart J. Biemond, Aart J. Nederveen

**Affiliations:** ^1^Department of Radiology and Nuclear Medicine, Amsterdam UMC, Location AMC, Amsterdam, Netherlands; ^2^Department of Biomedical Engineering and Physics, Amsterdam UMC, location AMC, Amsterdam Cardiovascular Sciences, Amsterdam, Netherlands; ^3^C.J. Gorter Center for High Field MRI, Department of Radiology, Leiden University Medical Center, Leiden, Netherlands; ^4^Leiden Institute for Brain and Cognition, Leiden University, Leiden, Netherlands; ^5^Institute of Medical Engineering, Graz University of Technology, Graz, Austria; ^6^Department of Hematology, Amsterdam UMC, Location AMC, Amsterdam, Netherlands; ^7^Division of Cardiology, Children’s Hospital Los Angeles, Keck School of Medicine, University of Southern California, Los Angeles, CA, United States

**Keywords:** vascular reactivity, sickle cell anaemia, arterial spin label (ASL) MRI, cerebral blood flow, cerebrovascular reactivity, hemodynamic abnormalities, arterial transit time

## Abstract

In patients with sickle cell disease (SCD), cerebral blood flow (CBF) is elevated to counteract anemia and maintain oxygen supply to the brain. This may exhaust the vasodilating capacity of the vessels, possibly increasing the risk of silent cerebral infarctions (SCI). To further investigate cerebrovascular hemodynamics in SCD patients, we assessed CBF, arterial transit time (ATT), cerebrovascular reactivity of CBF and ATT (CVR_*CBF*_ and CVR_*ATT*_) and oxygen delivery in patients with different forms of SCD and matched healthy controls. We analyzed data of 52 patients with severe SCD (HbSS and HbSβ^0^-thal), 20 patients with mild SCD (HbSC and HbSβ^+^-thal) and 10 healthy matched controls (HbAA and HbAS). Time-encoded arterial spin labeling (ASL) scans were performed before and after a vasodilatory challenge using acetazolamide (ACZ). To identify predictors of CBF and ATT after vasodilation, regression analyses were performed. Oxygen delivery was calculated and associated with hemoglobin and fetal hemoglobin (HbF) levels. At baseline, severe SCD patients showed significantly higher CBF and lower ATT compared to both the mild SCD patients and healthy controls. As CBF_*postACZ*_ was linearly related to CBF_*preACZ*_, CVR_*CBF*_ decreased with disease severity. CVR_*ATT*_ was also significantly affected in severe SCD patients compared to mild SCD patients and healthy controls. Considering all groups, women showed higher CBF_*postACZ*_ than men (*p* < 0.01) independent of baseline CBF. Subsequently, post ACZ oxygen delivery was also higher in women (*p* < 0.05). Baseline, but not post ACZ, GM oxygen delivery increased with HbF levels. Our data showed that baseline CBF and ATT and CVR_*CBF*_ and CVR_*ATT*_ are most affected in severe SCD patients and to a lesser extent in patients with milder forms of SCD compared to healthy controls. Cerebrovascular vasoreactivity was mainly determined by baseline CBF, sex and HbF levels. The higher vascular reactivity observed in women could be related to their lower SCI prevalence, which remains an area of future work. Beneficial effects of HbF on oxygen delivery reflect changes in oxygen dissociation affinity from hemoglobin and were limited to baseline conditions suggesting that high HbF levels do not protect the brain upon a hemodynamic challenge, despite its positive effect on hemolysis.

## Introduction

Sickle cell disease (SCD) is an inherited form of hemolytic anemia causing widespread organ damage including stroke (Rees et al., 2010; Debaun and Kirkham, 2016). Besides symptomatic stroke, SCD is also complicated by silent cerebral infarctions (SCI) which contribute to neuro-cognitive deficits (Strouse et al., 2009; Debaun and Kirkham, 2016). These SCI are found in the watershed areas of the cerebral arteries, probably due to diminished oxygen supply to the deep white matter (WM) structures (Dowling et al., 2010; Ford et al., 2018; Chai et al., 2019; Václavů et al., 2019). To counteract anemia and maintain oxygen delivery to the brain, resting cerebral blood flow (CBF) is elevated in patients with SCD (Numaguchi et al., 1990; Oguz et al., 2003; Bush et al., 2016). Unfortunately, this compensatory mechanism partially limits the ability to respond to hemodynamic stress, since arterioles and capillaries are already close to maximum vasodilation (Václavů et al., 2019), potentially leaving SCD patients at risk to acute changes in hemoglobin levels or nocturnal desaturation (DeBaun et al., 2012a). SCI are particularly common (38%) in patients with severe SCD, characterized by low hemoglobin levels and high CBF (Kwiatkowski et al., 2009; Ford et al., 2018), with a prevalence that increases with age. Although SCIs are also surprisingly common in patients with milder forms of SCD such as HbSC and HbSβ^+^-thalassemia (HbSβ^+^-thal), with a prevalence of SCIs in up to 37% of the patients (Zafeiriou et al., 2004; DeBaun et al., 2012a; Guilliams, 2015), little is known about cerebral hemodynamics in patients with milder forms of SCD.

A healthy vascular endothelium is essential to maintain cerebrovascular reactivity (CVR) to changes in oxygen demand or supply. Given the chronic vasodilatation and many endothelial stressors observed in SCD patients, the CVR has the potential to be developed into a biomarker for cerebrovascular health in these patients (Prohovnik et al., 1989, 2009; Hebbel et al., 2004; Nur et al., 2009; Kosinski et al., 2017). CVR has been studied extensively in SCD patients and is typically defined as the capacity of the blood vessels to dilate in response to a challenge such as breath-holding (Macedo-Campos et al., 2018), CO2 inhalation (Nur et al., 2009; Kosinski et al., 2017; Watchmaker et al., 2018) or administration of acetazolamide (ACZ) (Kedar et al., 2006; Václavů et al., 2019). Many MRI techniques can be used to quantify the resulting CVR changes including blood-oxygenation-level dependent (BOLD) (Davis et al., 1998; Hoge et al., 1999), phase-contrast (Patrick et al., 1996), and arterial spin labeling (ASL) MRI (Detre et al., 1999; Yen et al., 2002; Liu et al., 2019).

Arterial spin labeling has become a popular technique to measure CVR, because it provides absolute measurements of CBF during rest and under stress rather than just relative changes (Liu et al., 2019). To date, most ASL investigations of CVR have been limited to a single post-labeling delay (PLD), even though vasodilatory challenges are likely to change also the arterial transit time (ATT), defined as the duration of the labeled blood to flow from the labeling region to the tissue. With the introduction of time-encoded ASL, multiple ASL images with different post-labeling delays (PLD) can be acquired in a time efficient manner (Günther, 2007) to simultaneously calculate CBF and ATT. Not only does estimation of ATT improve the accuracy of CBF quantification (van Osch et al., 2018), ATT itself is increasingly being recognized as a useful additional hemodynamic biomarker (Mak et al., 2012; Al-Bachari et al., 2014; Paling et al., 2014). ATT is thought to be a measure of efficiency of blood supply, and has been used as a marker of large and small vessel health (Poublanc et al., 2013; Federau et al., 2017). This could be especially valuable in patients with SCD, as SCD is known to affect both large and small blood vessels. In SCD, ATT was found to be reduced compared to healthy controls (Juttukonda et al., 2017; Kawadler et al., 2018) under resting conditions. However, no studies to date have reported ATT changes in response to a cerebral vasodilatory challenge.

In this work, we studied patients with severe and milder forms of SCD and matched healthy controls, in order to provide a comprehensive overview of how cerebral hemodynamics differ across a large range of disease severity of SCD. Using time-encoded ASL, we measured CBF, ATT, and oxygen delivery at rest and following ACZ administration to calculate CVR_*CBF*_ and CVR_*ATT*_. We hypothesized that patients with milder forms of SCD would show affected cerebral hemodynamics compared to healthy controls, but to a lesser extent than patients with severe SCD. All measured and derived hemodynamic parameters were compared with the laboratory and demographic predictors to determine which factors influence cerebral vasodilatory capacity.

## Materials and Methods

### Demographics

The study was approved by the local Institutional Review Boards at the Amsterdam University Medical Centers in The Netherlands and at Children’s Hospital Los Angeles in the United States. In both institutions, the study was performed in accordance with the Declaration of Helsinki and written informed consent was obtained from all participants. In total, 56 patients with severe SCD (HbSS and HbSβ^0^-thal), 20 patients with the milder SCD phenotype (HbSβ^+^-thal and HbSC) and 12 healthy age and ethnicity matched controls (HbAA and HbAS) were recruited between 2018 and 2020. Exclusion criteria were contraindications to MRI or ACZ, pregnancy or breastfeeding, history of cerebral pathology that compromises measurements, such as vasculopathy, cerebral palsy, brain tumor, meningitis or overt infarct, sickle cell crisis at the moment of the participation and hospitalization 1 month before the study day. Patients that were treated with chronic transfusions were examined up to 2 days before their routine transfusion.

### Laboratory Markers

Prior to the MRI examination, blood was drawn from the upper extremity vein to determine hemoglobin, hematocrit, fetal hemoglobin (HbF) and markers of hemolysis such as total bilirubin and reticulocytes.

### Image Acquisition

All experiments were performed on a Philips 3T MRI scanner (Philips Medical Systems, Best, Netherlands), using a 32-channel receive head-coil and body-coil transmission. Time-encoded pseudo-continuous ASL (pCASL), M0 and 2D phase-contrast scans were acquired prior to and 10 min after ACZ administration (16 mg/kg i.v., max 1400 mg). During ACZ infusion, a 1 mm isotropic T1 weighted anatomical scan was acquired. For ASL acquisitions, a 2D Echo Planar Imaging Time-encoded pCASL sequence with the following parameters was used: TR/TE = 5040/16 ms; Hadamard-8 matrix with seven blocks of 2000, 800, 500, 300, 250, and 150 ms; PLD = 100 ms; SENSE = 2.5; voxel size = 3×3×6 mm^3^; FOV = 240×240×114 mm^3^; 2 FOCI background suppression pulses; SPIR fat suppression; NSA = 12; total scan time 8:41 min. The ASL imaging volume was placed parallel to the anterior commissure – posterior commissure (AC-PC) plane. The labeling plane was positioned 9 cm below the center of the imaging plane avoiding labeling at siphons. M0 scans were acquired using the same imaging parameters except for the TR, which was 2500 ms, and switching off labeling and background suppression. Blood velocity in the main feeding arteries was derived from phase contrast scans which were acquired with a single slice 2D Fast Field Echo (FFE) readout, FOV of 220×220 mm^2^, voxel size of 0.6 × 0.6 mm^2^, slice thickness of 5 mm, TR/TE of 22/14 ms, flip angle of 10° and velocity encoding (VENC) of 80 cm/s.

### Post Processing

#### T1 Weighted Anatomical Scans

T1 weighted anatomical scans were segmented into gray matter (GM) and WM tissue probability maps, denoted as pGM and pWM, using CAT12 (Gaser and Dahnke, 2016). pGM maps were coregistered to the CBF image and the resulting deformation fields were applied to pWM maps as well. Subsequently, GM and WM masks were obtained by thresholding the pGM and pWM maps with 0.7 and 0.9, respectively.

#### ASL Scans

Arterial spin labeling scans were motion corrected and coregistered to the baseline ASL scan using SPM12 (Wellcome Trust Center for Neuroimaging, London, United Kingdom). Subsequently, the ASL images were subtracted according to the Hadamard-8 matrix to obtain seven perfusion weighted images (PWI) effectively corresponding to PLDs of 100, 250, 450, 700, 1000, 1500 and 2300 ms. The PWI time series were denoised using a spatio-temporal total generalized variation (TGV) algorithm as described previously (Spann et al., 2017). As background suppression pulses were applied within the labeling duration, different PLD images were not preceded by the same number of background suppression pulses. Hence, the different PLD images were corrected individually by a factor of 0.95^*n*^ in which n is the number of applied background suppression pulses during the PLD. Quantification of the seven PWIs was performed using the FSL based BASIL toolbox which uses Bayesian inference to fit CBF, ATT, and arterial blood volume fraction in a voxelwise manner, according to the extended kinetic model (Chappell et al., 2010). By including a macrovascular component in the model, this approach enables isolating the intravascular signal. Thus, ATT describes the arrival time in the tissue rather than the macrovasculature. We used group-based longitudinal relaxation times (T1) for arterial blood of 1818 ms for patients (Vaclavu et al., 2016). For controls, a hematocrit derived blood T1 was used (Lu et al., 2004). Subject specific labeling efficiency were calculated based on the flow weighted mean velocity measured (Aslan et al., 2010). Before creating responsivity maps, Gaussian smoothing (FWHM = 3.5 mm) was applied to CBF and ATT maps within GM and WM separately, to avoid mixing of GM and WM estimates. CVR_*CBF*_ maps were created from the pre- and post ACZ CBF maps according to:

$$C\,V\,R_{C\,B\,F}=\left(C\,B\,F_{p\,o\,s\,t\,A\,C\,Z}-C\,B\,F_{p\,r\,e\,A\,C\,Z}\right)/\left(C\,B\,F_{p\,r\,e\,A\,C\,Z}\right)*100\%$$

Similarly, CVR_*ATT*_ maps were created from pre- and post ACZ ATT maps according to:

$$C\,V\,R_{A\,T\,T}=\left(A\,T\,T_{p\,o\,s\,t\,A\,C\,Z}-A\,T\,T_{p\,r\,e\,A\,C\,Z}\right)/\left(A\,T\,T_{p\,r\,e\,A\,C\,Z}\right)*100\%$$

Oxygen delivery in GM and WM was calculated as a product of CBF and oxygen content. Oxygen content was calculated using the following equation:

$$O\,x\,y\,g\,e\,n\,C\,o\,n\,t\,e\,n\,t=1.34*H\,e\,m\,o\,g\,l\,o\,b\,i\,n*S\,p\,O_{2}+0.0031*p\,O_{2}$$

In which 1.34 is a constant representing the amount of oxygen that can bind to hemoglobin, hemoglobin is the patient-specific hemoglobin, SpO2 is the arterial oxygen saturation, which is assumed to be 0.98 in all participants, 0.0031 is the solubility coefficient of oxygen in human plasma and pO2 is arterial oxygen tension, which is assumed to be 100 mmHg for room air.

#### Phase Contrast Scans

Regions of interest were manually drawn in ITK snap on all main brain feeding arteries captured by the 2D imaging slice. The number of included arteries varied per participant due to differences in anatomy. The flow weighted mean of all arteries combined was calculated as:

$$F\,l\,o\,w\,w\,e\,i\,g\,h\,t\,e\,d\,m\,e\,a\,n\,v\,e\,l\,o\,c\,i\,t\,y=s\,u\,m\,\left(V^{2}\right)/s\,u\,m\,\left(V\right)$$

in which V is the observed velocity in each individual voxel. The flow weighted mean velocity was used to derive subject specific labeling efficiencies for CBF quantification.

### Statistical Analysis

Statistical analysis was performed in SPSS (IBM, NY, United States). Analysis of variance (ANOVA) (or Kruskal Wallis in case of non-normality) was used to compare the imaging and laboratory parameters between different groups. In case of multiple comparisons, significance level was adjusted accordingly using Sidak multiple comparison correction. Paired-samples *t*-tests were used to test the differences in imaging parameters before and after ACZ administration. To study trends in CVR across the groups, the Jonckheere–Terpstra trend test was used. Associations between imaging measures and the markers of anemia and hemolysis were studied using correlation analysis. Because CVR_*CBF*_ and CVR_*ATT*_ were calculated as the ratio of two values, they are nonlinear and non-Gaussian distributed, inhibiting the use of linear statistics. Therefore, post ACZ values were used to identify predictors for CBF and ATT in response to a vasoactive stimulus. First, CBF_*postACZ*_ and ATT_*postACZ*_ were corrected for CBF_*preACZ*_ and ATT_*preACZ*_, respectively, by performing a simple linear regression and saving the residuals. Then, stepwise multiple regression analysis was performed between the residuals and the following predictors: age, sex, participant groups, hemoglobin, total bilirubin, reticulocytes, and HbF, using pairwise deletion to account for missing values. Variables with *p* < 0.05 were retained in the multiple regression model as a significant predictor for CBF_*postACZ*_ or ATT_*postACZ*_.

## Results

Out of 56 severe SCD patients and 12 healthy controls recruited, respectively, 4 and 2 were excluded from analysis due to aborted ASL scans, data corruption, large CBF asymmetry (suspicion of vasculopathy or labeling artifacts) and unresolved errors during post-processing. Participant characteristics of the remaining 52 severe SCD patients, 20 mild SCD patients and 10 healthy controls are shown in Table 1. No significant differences were found in age and sex between the groups. Hemoglobin genotypes were as follows: severe SCD; 44 HbSS and 8 Hbβ^0^, mild SCD; 10 HbSC and 10 Hbβ^+^ and controls: 6 HbAA and 4 HbAS (Supplementary Table 1). Statistically significant differences were observed in hemoglobin, hematocrit, total bilirubin and reticulocytes between the groups. None of the mild SCD patients was treated with chronic transfusion therapy. Four of the severe SCD patients received chronic exchange transfusion, three received chronic simple transfusion. The average time between the last transfusion and the day of study participation was 27 ± 5 days. Patient characteristics per treatment group are shown in Supplementary Table 2.

**TABLE 1:** Demographic information (mean±std) of the participants and test statistics from ANOVA (F) with post-hoc Tukey test and Kruskal-Wallis (H) test with post-hoc Dunn test.

	Severe SCD	Mild SCD	Controls	Test statistics (*p*-value)	*p*-value (S-C)*	*p*-value (S-M)*	*p*-value (M-C)*
**Patient characteristics**							
N	52	20	10	−			
Age (y)	29 ± 10	33 ± 12	27 ± 6	F: 1.96 (0.15)	0.57	0.09	0.10
Sex (Women/Men)	22/30	6/14	5/5	X ^2^: 1.36 (0.51)	0.74	0.42	0.43
**Blood markers**							
Hemoglobin (g/dL)	8.8 ± 1.2	11.4 ± 1.5	13.3 ± 1.6	H: 41.4 (<0.01)	<0.01 †	<0.01 †	0.14†
Hematocrit (%)	25.5 ± 3.9	33.6 ± 4.3	40.2 ± 4.1	H: 43.1 (<0.01)	<0.01 †	<0.01 †	0.11†
Total Bilirubin (mg/dL)	3.4 ± 2.1	1.7 ± 0.8	0.5 ± 0.3	H: 21.5 (<0.01)	<0.01 †	<0.01 †	0.09†
Reticulocytes (10e9/L)	270.6 ± 131.5	129.4 ± 54.3	94.5 ± 48.3	H: 26.9 (<0.01)	<0.01 †	<0.01 †	0.39†
Hydroxyurea (N)	25 (52%)	9 (45%)	0 (0%)	−	−	0.82	−
Chronic transfusions (N)	7 (13%)	0 (0%)	0 (0%)	−	−	−	−

*post-hoc statistics: S-C = severe SCD compared to controls, S-M = severe SCD compared to mild SCD, M-C = mild SCD compared to controls. F, X ^2^ and H denote test statistics from ANOVA, Chi square and Kruskal-Wallis tests, respectively. “†” indicate p-values from non-parametric tests. P-values are shown without multiple comparison correction. Significance level was corrected to 0.017 using Sidak multiple comparison correction. Bold + underlined values denote statistically significant p-values after multiple comparison correction.

**FIGURE 1:**
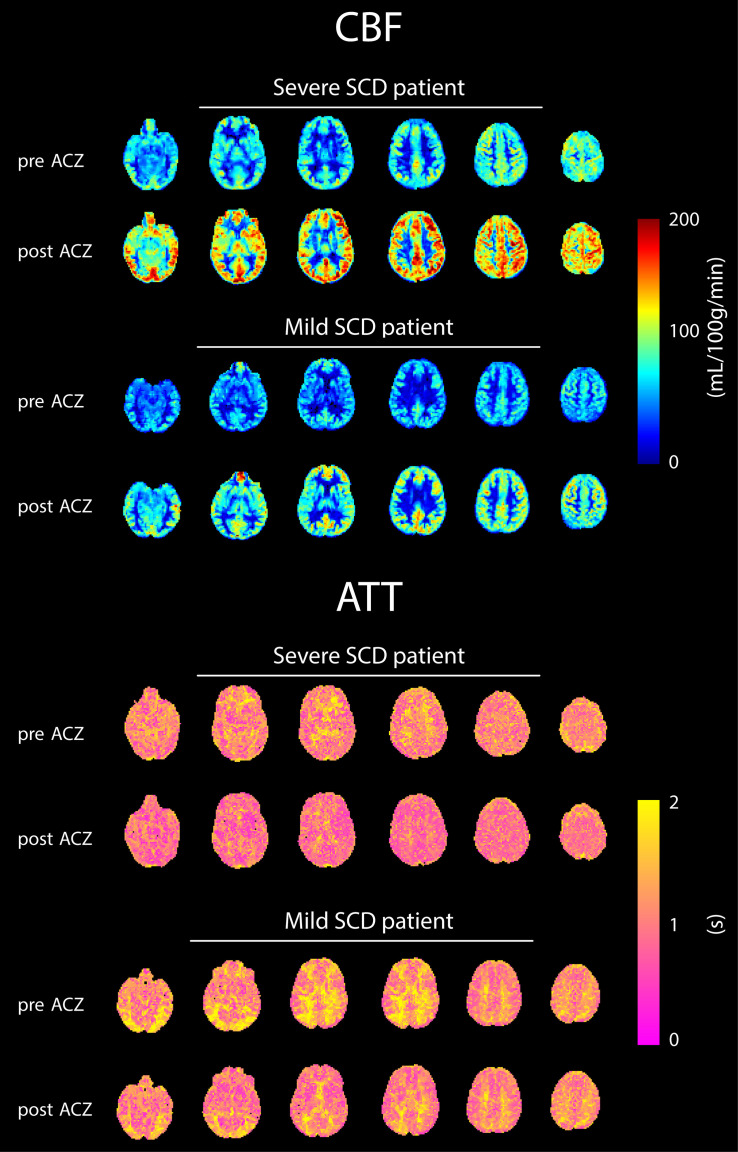
Six axial slices of CBF and ATT maps before and after ACZ administration of representative patients with severe and mild SCD.

Representative CBF and ATT maps from a severe and mild SCD patient, before and after ACZ administration, are shown in Figure 1. An increase in CBF and a decrease in ATT can be observed in GM and WM regions after ACZ administration. GM and WM CBF were significantly higher, both before and after ACZ administration, in patients with severe SCD compared to patients with mild SCD as well as compared to controls (Table 2 and Supplementary Figure 1). After ACZ administration, GM and WM CBF significantly increased in all groups (*p* < 0.01). Similarly, GM and WM ATT were significantly shorter after ACZ administration in all groups (*p* < 0.01). Moreover, baseline ATT was significantly shorter in severe SCD patients compared to patients with mild SCD and compared to controls in both GM and WM. The same was observed after ACZ administration, except for GM ATT between patients with severe SCD and controls. No significant differences in CBF and ATT were found between patients with mild SCD and controls nor between patients receiving hydroxyurea, chronic transfusion treatment or neither of these treatments (Supplementary Table 2).

**TABLE 2:** CBF and ATT before and after ACZ administration and CVR_*CBF*_ and CVR_*ATT*_ [median (interquartile range)] per participant group with *p*-values between groups.

	Severe SCD		Mild SCD		Controls		*p*-value (S-C)*		*p*-value (S-M)*		*p*-value (M-C)*	
						
CBF and ATT	Pre ACZ	Post ACZ	Pre ACZ	Post ACZ	Pre ACZ	Post ACZ	Pre ACZ	Post ACZ	Pre ACZ	Post ACZ	Pre ACZ	Post ACZ
GM CBF (mL/100g/min)	84.0 [23.0]	107.2 [27.0]	57.9 [12.1]	80.3 [29.4]	53.7 [14.9]	79.3 [29.6]	**<0.01**	**<0.01**	**<0.01**	**<0.01**	0.30	0.68
WM CBF (mL/100g/min)	46.1 [14.5]	60.0 [20.8]	31.4 [10.3]	41.0 [17.1]	30.6 [7.3]	40.5 [15.7]	**<0.01†**	**<0.01†**	**<0.01†**	**<0.01†**	0.39**†**	0.40**†**
GM ATT (s)	0.96 [0.11]	0.87 [0.08]	1.08 [0.10]	0.94 [0.04]	1.09 [0.12]	0.95 [0.13]	**<0.01†**	0.02†	**<0.01†**	**<0.01†**	0.85†	0.52†
WM ATT (s)	1.06 [0.14]	0.96 [0.13]	1.19 [0.15]	1.06 [0.06]	1.26 [0.16]	1.05 [0.23]	**<0.01†**	**0.01†**	**<0.01†**	**<0.01†**	0.90†	0.64†
**CVR_*CBF*_ and CVR_*ATT*_**												
GM CVR_*CBF*_ (%)	29.1 [24.0]		40.1 [40.1]		48.6 [35.0]		**<0.01**		0.02		0.29	
WM CVR_*CBF*_ (%)	29.1 [28.1]		34.8 [34.6]		39.4 [39.0]		0.18†		0.45†		0.51†	
GM CVR_*ATT*_ (%)	−4.3 [7.3]		−9.7 [9.5]		−10.6 [10.6]		**<0.01**		**<0.01**		0.90	
WM CVR_*ATT*_ (%)	−6.6 [6.6]		−8.8 [9.0]		−11.6 [11.0]		0.02		**<0.01**		0.84	

*post-hoc statistics: S-C = severe SCD compared to controls, S-M = severe SCD compared to mild SCD, M-C = mild SCD compared to controls. “†” indicate *p*-values from non-parametric tests. *P*-values are shown without multiple comparison correction. Significance level was corrected to 0.017 using Sidak multiple comparison correction. Bold + underlined values denote statistically significant p-values after multiple comparison correction.

**FIGURE 2:**
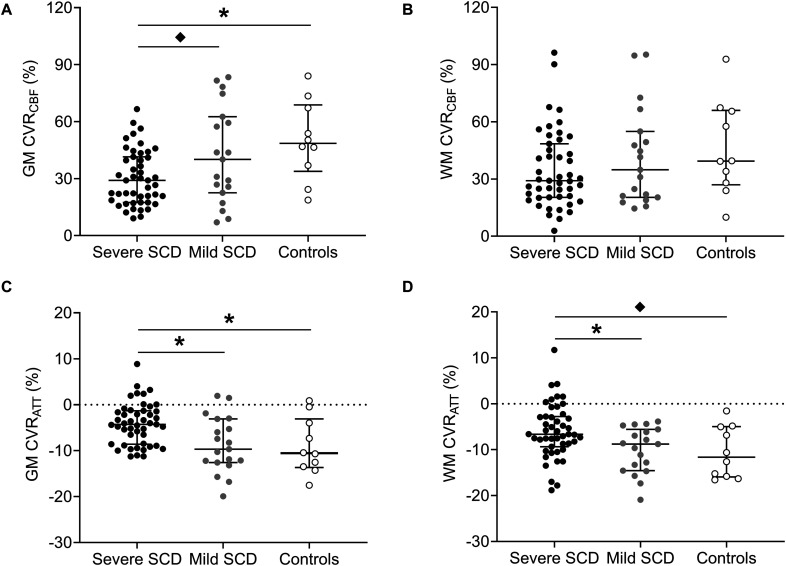
Dot plots with median and interquartile ranges showing CVR_*CBF*_ and CVR_*ATT*_ in GM **(A,C)** and WM **(B,D)** in patients with severe- and mild SCD and healthy controls. Statistically significant differences, after multiple comparison correction, are indicated by lines with asterisks (*p* < 0.017). “♦” denote statistically significant differences that did not remain after multiple comparison correction.

In patients with severe SCD, GM, but not WM, CVR_*CBF*_ was significantly lower compared to controls (Figures 2A,B and Table 2). GM CVR_*CBF*_ in severe patients was also lower compared to mild patients, but this did not remain statistically significant after multiple comparison correction. GM CVR_*ATT*_ was significantly higher in patients with severe SCD compared to patients with mild SCD and compared to controls, whereas for WM CVR_*ATT*_, only the significant difference between the patient groups (Figures 2C,D) remained after multiple comparison correction. These differences between groups were also observed visually from the group averaged CVR maps (Supplementary Figure 2). Patients with mild SCD were less affected compared to severe SCD patients, resulting in a statistically significant linear trend between the groups in GM CVR_*CBF*_ (J-T = 2.87; *p* < 0.01), GM CVR_*ATT*_ (J-T = −3.39; *p* < 0.01), and WM CVR_*ATT*_ (J-T = −2.87; *p* < 0.01). CVR_*CBF*_ varied inversely with CVR_*ATT*_ both in GM and WM (GM: *r*^2^ = 0.32; *p* < 0.01 and WM: *r*^2^ = 0.20; *p* < 0.01). CVR_*CBF*_ was significantly higher in women compared to men both in GM and WM (GM: *p* = 0.01 and WM: *p* < 0.01), however, CVR_*ATT*_ did not show sex differences (GM: *p* = 0.40 and WM: *p* = 0.07). We did not observe differences in CVR_*CBF*_ and CVR_*ATT*_ between patients receiving hydroxyurea, chronic transfusion therapy, or no prophylactic therapy (Supplementary Tables 2–4).

**FIGURE 3:**
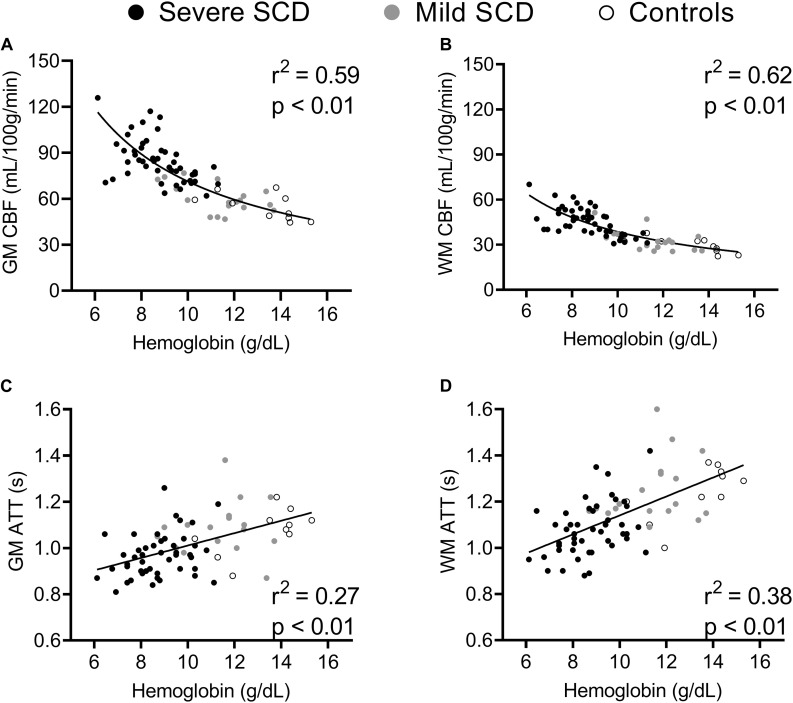
Scatterplots displaying correlations between hemoglobin and GM and WM CBF **(A,B)** and GM and WM ATT **(C,D)** at baseline.

Baseline CBF was inversely related to hemoglobin (CBF ∼ 1/Hb) in both GM and WM (Figures 3A,B). Spearman’s rank-order correlation showed that GM and WM CBF_*preACZ*_ increased with total bilirubin (GM: *r*^2^ = 0.30; *p* < 0.01 and WM: *r*^2^ = 0.18; *p* < 0.01) and reticulocytes (GM: *r*^2^ = 0.17; *p* < 0.01 and WM: *r*^2^ = 0.09; *p* < 0.01), not shown. GM and WM ATT_*preACZ*_ increased linearly with hemoglobin (Figures 3C,D) and were negatively related to total bilirubin (GM: Spearman’s *r*^2^ = 0.15; *p* < 0.01 and WM: Spearman’s *r*^2^ = 0.15; *p* < 0.01) and reticulocytes (GM: Spearman’s *r*^2^ = 0.12; *p* < 0.01 and WM: Spearman’s *r*^2^ = 0.11; *p* < 0.01). Stepwise multiple regression showed that after correcting for 1/Hb, age and HbF were statistically significant predictors for GM CBF_*preACZ*_ (Age; β = −0.39, *p* < 0.01. HbF; β = 0.27, *p* = 0.025) but not for WM CBF_*preACZ*_.

CBF_*postACZ*_ and ATT_*postACZ*_ were linearly related to, respectively, CBF_*preACZ*_ and ATT_*preACZ*_ in GM (*r*^2^ = 0.65; *p* < 0.01 and *r*^2^ = 0.69; *p* < 0.01, respectively) and WM (*r*^2^ = 0.72; *p* < 0.01 and *r*^2^ = 0.72; *p* < 0.01, respectively) (Supplementary Figure 3). After correcting for resting conditions, stepwise multiple regression was performed including all participant groups. Sex was the only significant predictor for GM CBF_*postACZ*_ with women showing higher GM CBF_*postACZ*_ (*r*^2^ = 0.16; *p* < 0.01). Sex and HbF were statistically significant predictors for WM CBF_*postACZ*_ (Sex; β = 0.33, *p* = 0.01, HbF; β = −0.25, *p* = 0.045). Women also demonstrated shorter GM and WM ATT_*postACZ*_ (*r*^2^ = 0.07; *p* = 0.026 and *r*^2^ = 0.12; *p* < 0.01, respectively) than men after controlling for pre stimulus conditions. An alternative analysis, investigating predictors for ΔCBF and ΔATT, resulted in similar results and is shown in the Supplementary Materials.

**FIGURE 4:**
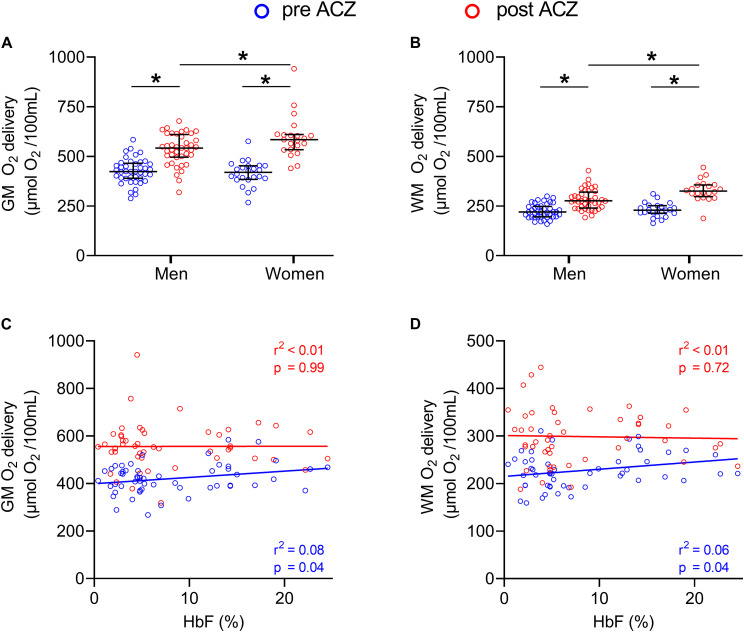
GM and WM oxygen delivery before and after ACZ administration. Dot plots showing GM and WM oxygen delivery in men and women before and after ACZ administration **(A,B)** and scatter plots with correlations between HbF and GM and WM oxygen delivery in patients **(C,D)**. Error bars denote median and interquartile range, horizontal bars with asterisks denote statistically significant differences (*p* < 0.05).

No significant differences were found in baseline oxygen delivery between the groups (GM: *F* = 1.78; *p* = 0.18 and WM: *F* = 0.17; *p* = 0.84). After ACZ administration, GM and WM oxygen delivery significantly increased in both patient groups and controls (*p* < 0.01) but no significant differences were found between the groups (GM: *F* = 1.79; *p* = 0.18, WM: *F* = 0.49; *p* = 0.62). Baseline GM and WM oxygen delivery were not significantly different between men and women (*p* = 0.28 and *p* = 0.43, respectively) and significantly increased after ACZ administration in men and women (*p* < 0.01) (Figures 4A,B). After ACZ administration, significantly higher oxygen delivery in GM and WM were observed in women compared to men (GM: *p* = 0.042, WM: *p* < 0.01). In patients (severe and mild), GM, but not WM, oxygen delivery was positively associated with HbF under resting conditions, but this relation disappeared following ACZ (Figures 4C,D).

## Discussion

In this study, we demonstrated that during a vasodilatory challenge CBF and WM ATT are mainly determined by the baseline vascular state (flow and transit time). CVR_*CBF*_ and CVR_*ATT*_ were increasingly affected by, genotype based, disease severity, which was explained by its effect on baseline CBF and ATT. However, we did identify two novel modulators of ACZ response. Firstly, women exhibited more robust increases in CBF and oxygen delivery as well as shortening of WM ATT in response to ACZ after correcting for baseline cerebrovascular dynamics. Secondly, HbF levels were associated with increased resting CBF and oxygen delivery in GM, but had no effect on peak oxygen delivery. We also demonstrated that ATT consistently shortened with ACZ in both the GM and WM allowing the definition of a CVR_*ATT*_ that was highly correlated with CVR_*CBF*_.

The dependence of CVR_*CBF*_ on resting CBF arises from the linear relationship between pre- and post ACZ flow. We observed a constant increase of flow with ACZ, independent of the baseline CBF. As a result, the calculated CVR declines reciprocally with baseline CBF. Differences in baseline CBF between genotypic SCD groups reflect differences in hemoglobin levels and HbF percentages. Lower hemoglobin levels are thought to be compensated by an increase in baseline CBF to maintain normal oxygen delivery to the brain. This effect leads to significantly higher baseline CBF and shorter ATT in patients with severe SCD compared to patients with mild SCD and healthy controls. After controlling for hemoglobin level, baseline GM CBF increased with HbF percentage, most likely to compensate for the higher oxygen affinity of HbF (Wood, 2019). Thus, the effect of disease severity on post ACZ measurements and CVR are primarily apparent through its effects on hemoglobin and HbF percentage which ultimately affect baseline conditions.

We did not observe any saturation of vasodilatory response as has been suggested in previous studies (Prohovnik et al., 2009; Kosinski et al., 2017; Václavů et al., 2019). This could be because our study included a significant fraction of patients with milder disease and a limited number of patients with baseline GM CBF values higher than 100 mL/100/min. Another potential difference with previous ASL studies is that time-encoded ASL appropriately corrects for the shortening in ATT with ACZ stimulation, leading to more accurate CBF measurements. In single delay time ASL measurements, post ACZ CBF may be underestimated since ATT changes are not taken into account or globally estimated with a separate scan (Václavů et al., 2019), ultimately leading to an underestimation of CVR_*CBF*_.

In addition to baseline CBF, sex was a strong predictor of the response to ACZ. Women showed a more robust response to the vasodilatory challenge than men, suggesting a better cerebrovascular reactivity. Moreover, these results persisted after correcting for hemoglobin levels, indicating that these differences are not related to possible sex differences in hemoglobin levels. Together with significantly higher oxygen delivery in women compared to men after ACZ administration, these data suggest that women are better capable of increasing oxygen delivery to the brain under stress. Although the effect sizes observed translate to clinically meaningful differences, future studies should investigate sex effects in white matter disease in relation to cognitive outcomes, as the current dataset is underpowered to do so. Nevertheless, these preliminary results could explain why SCIs are less frequently observed in female compared to male SCD patients (DeBaun et al., 2012b; van der Land et al., 2016). We aim to further investigate the relation between vascular reactivity and SCI prevalence in SCD patients in our future work.

We also observed HbF levels to be an important modulator of cerebrovascular dynamics. In patients with severe SCD, hemoglobin levels were positively associated with HbF concentration. Given the reciprocal relationship between hemoglobin and CBF, we expected that high HbF would tend to decrease resting CBF. However, we observed that HbF concentration and GM CBF were positively related for any given hemoglobin level, thereby improving the calculated GM oxygen delivery at baseline. We hypothesize that this phenomenon is caused by the left shift of the hemoglobin dissociation curve of HbF, potentially lowering cerebral oxygen extraction (Fields et al., 2019; Wood, 2019) for any given partial pressure of oxygen in the brain. With lower oxygen extraction, higher resting CBF would be required to maintain normal oxygen exchange between the blood and brain tissue.

Interestingly, post ACZ cerebral oxygen delivery was independent of HbF, suggesting that HbF does not increase the maximum oxygen delivery to the brain; in fact, the rise in GM oxygen delivery with ACZ decreased with HbF concentration. Thus, the beneficial effects of HbF (higher hemoglobin, higher resting oxygen delivery, lower oxygen extraction fraction) (Fields et al., 2019) might have been counterbalanced by a decrease in recruitable oxygen delivery under vasodilator stress. Simply stated, the brain’s ability to recruit additional oxygen through extraction and vasodilation may be coupled through the hemoglobin dissociation curve (Wood, 2019). Thus, modulators of the p50 of hemoglobin may cause trade-offs in flow and extraction reserve.

In this study, we have also shown that ACZ administration consistently shortens ATT in GM and WM. We also presented CVR_*ATT*_ as a novel measure of vascular reactivity in SCD patients. CVR_*ATT*_ was closely correlated with the traditional CVR_*CBF*_, exhibited similar predictors (except HbF) and was similar in GM and WM. In contrast to CVR_*CBF*_, CVR_*ATT*_ was significantly different between patients with mild and severe SCD in WM. This might be explained by the fact that less ASL signal is required to determine ATT than to accurately quantify CBF. This could indicate that CVR_*ATT*_ might be able to discriminate more subtle impairments in cerebrovascular reactivity than CVR_*CBF*_. Therefore, we believe that CVR_*ATT*_ could potentially act as a complementary biomarker to CVR_*CBF*_, especially in WM, where ASL signal is low, but this hypothesis requires a confirmatory study, which also includes the presence of ATT in the CBF quantification.

Some limitations of this study have to be acknowledged. First, it remains challenging to measure CBF in WM by ASL. Even though patients with SCD have increased blood flow and longer blood T1, WM ASL signal suffers from low SNR. This may explain why we did not observe differences in WM CVR_*CBF*_ between the groups nor in WM CVR_*ATT*_ between severe SCD patients and controls. Secondly, we did not estimate the T1 of blood on individual basis for patients, but instead we used group-based T1 values for patients with SCD as recommended by Vaclavu et al. (2016). Although this avoids CBF overestimation due to the longer blood T1 in patients with SCD on a group basis, it is still less accurate than subject-specific measurements, albeit with higher precision. Thirdly, our comparisons between the participant groups were significantly hampered by a mismatch in group size (*n* = 52, *n* = 20 and *n* = 10). This might explain why we did not find statistically significant differences in GM CVR_*CBF*_ and GM CVR_*ATT*_ between the mild SCD group and healthy controls. This mismatch might also have further complicated comparisons between WM CVR_*ATT*_ of severe SCD patients and controls. Lastly, the effects of prophylactic therapy might have affected our results. However, because patients receiving hydroxyurea therapy, which increases HbF, were similarly represented in both groups (52% and 45%) and patients were scanned at the end of their transfusion cycle, we believe that this effect was minimal.

This study provides a comprehensive overview of differences in cerebral hemodynamics in patients with SCD, reporting for the first time on CBF, ATT, CVR_*CBF*_, CVR_*ATT*_, and oxygen delivery in SCD patients with different genotypes. We found that vasodilation in response to ACZ is mainly determined by baseline CBF and ATT while there is no other added effect of disease severity. After correcting for baseline CBF and ATT effects, sex showed to be the dominant factor for vasodilation. Subsequently, women showed higher CVR_*CBF*_ and oxygen delivery in response to ACZ compared to men. We hypothesize that these preliminary results could explain the lower prevalence of silent cerebral infarcts in women compared to men. Lastly, HbF did not contribute to increased oxygen delivery to the brain in response to ACZ, which may suggest that HbF does not protect SCD patients from brain damage under acute conditions.

## Data Availability Statement

The datasets presented in this article are not readily available because this data represents an interim data analysis from NCT0371592, which is open for enrollment until 12/31/2022. The complete data set from this trial will be made available upon reasonable request after that time with an approved data sharing agreement. Requests to access the datasets should be directed to AN, a.j.nederveen@amsterdamumc.nl.

## Ethics Statement

The studies involving human participants were reviewed and approved by the Institutional Review Board at the Amsterdam University Medical Centers and Children’s Hospital Los Angeles in the United States. Written informed consent was provided by the participants’ legal guardian/next of kin in case of minors (<18yo) participating to the study.

## Author Contributions

LA-H and KB involved in data acquisition, data analysis, administrative duties, and writing the manuscript. BC, AS, and JW contributed to this work by assisting with data analysis, writing the manuscript, and designing figures. MO, SS, and EN contributed to this work by helping to set up this research and reviewing the manuscript before submission. BB and AN lead the supervision team of both first authors and were involved in all activities and decisions during the research. AN is last author of this manuscript. All authors contributed to the article and approved the submitted version.

## Conflict of Interest

The authors declare that the research was conducted in the absence of any commercial or financial relationships that could be construed as a potential conflict of interest.

## Supplementary Material

Supplementary Figure 1 Dot plots with median + interquartile ranges showing CBF **(A,B)** and ATT **(C,D)** in GM and WM, before and after ACZ administration in all participant groups. “*” denote statistically significant differences after multiple comparison correction compared to corresponding pre- or post-contrast condition in severe SCD group (*p* < 0.017). “♦” denote statistically significant differences that did not remain after multiple comparison correction. No such significant differences were found between mild SCD and controls. All measures changed significantly from pre- to post ACZ within groups (not indicated).

Supplementary Figure 2 Group-averaged CVR_*CBF*_ and CVR_*ATT*_ maps in GM and WM of healthy controls, mild – and severe patients with SCD.

Supplementary Figure 3 Scatterplots displaying correlations between CBF_*preACZ*_ and CBF_*postACZ*_ and between ATT_*preACZ*_ and ATT_*postACZ*_ in GM and WM.

Supplementary Table 1 Hb types per subject group. For clarity, patients receiving chronic transfusion therapy were not included.

Supplementary Table 2 Demographic information (mean±std) and hemodynamic parameters (median [IQ range]) of patients, divided by treatment group, and test statistics from ANOVA (F) test, Chi square (X ^2^) and Kruskal-Wallis (H). *P*-values are shown without multiple comparison correction. Significance level was corrected to 0.017 using Sidak multiple comparison correction. *P*-values that remain statistically significant after multiple comparison correction are underlined and shown in bold.

Supplementary Table 3 Demographic information (mean±std) and hemodynamic parameters (median [IQ range]) of severe SCD patients only, divided by treatment group, and test statistics from ANOVA (F) test, Chi square (X ^2^) and Kruskal-Wallis (H). *P*-values are shown without multiple comparison correction. Significance level was corrected to 0.017 using Sidak multiple comparison correction. *P*-values that remain statistically significant after multiple comparison correction are underlined and shown in bold.

Supplementary Table 4 Demographic information (mean±std) and hemodynamic parameters (median [IQ range]) of mild SCD patients only, divided by treatment group. Statistically significant *p* values are underlined and shown in bold.
