# Supplementary figures and images for: Impairment of Cerebrovascular Hemodynamics in Patients With Severe and Milder Forms of Sickle Cell Disease
Source: Front Physiol. 2021 Apr 20;12:645205. doi: 10.3389/fphys.2021.645205 (PMC8093944; doi:10.3389/fphys.2021.645205)

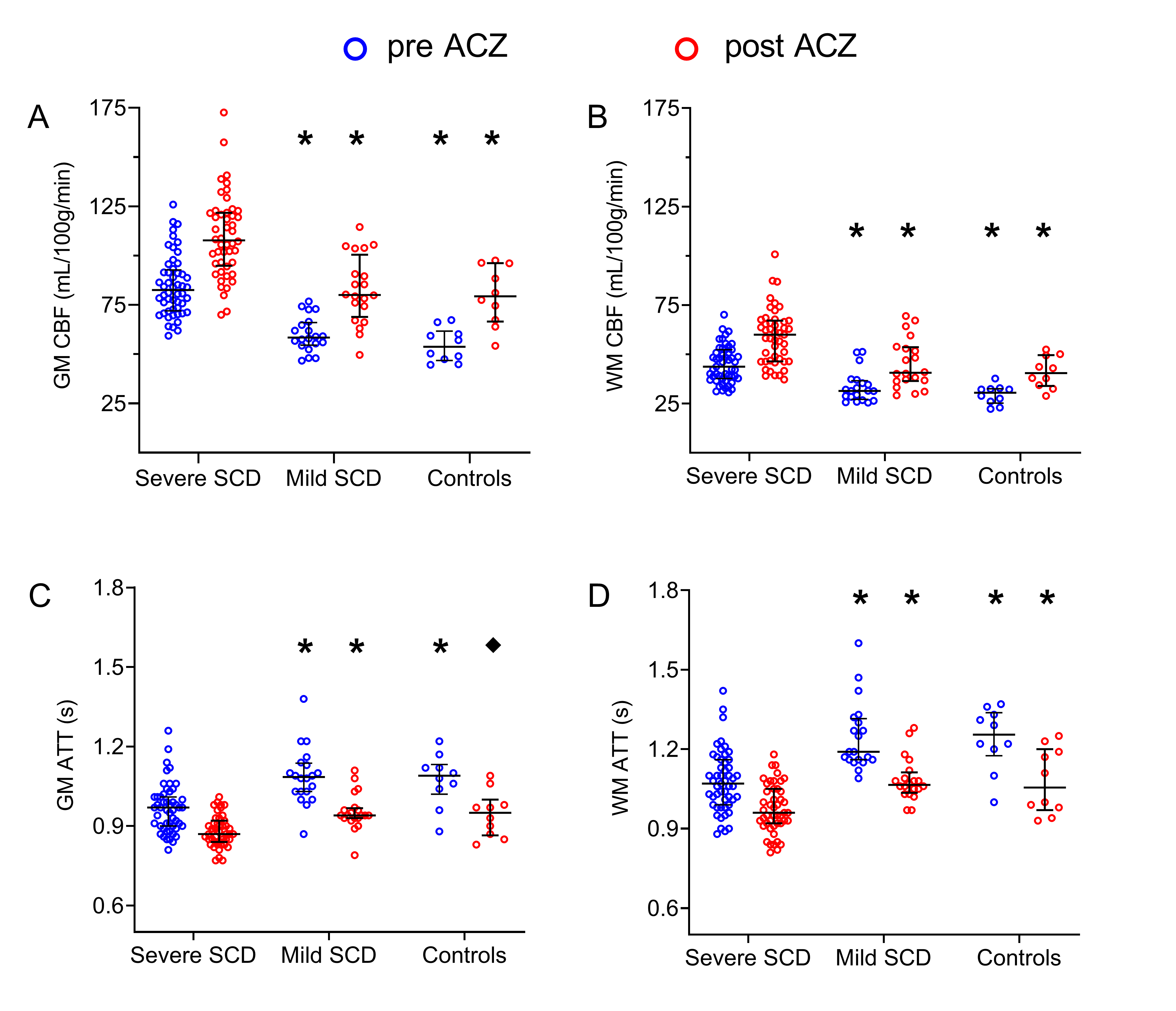

Supplement: Supplementary Figure 1 — Dot plots with median + interquartile ranges showing CBF (A,B) and ATT (C,D) in GM and WM, before and after ACZ administration in all participant groups. “*” denote statistically significant differences after multiple comparison correction compared to corresponding pre- or post-contrast condition in severe SCD group (p < 0.017). “♦” denote statistically significant differences that did not remain after multiple comparison correction. No such significant differences were found between mild SCD and controls. All measures changed significantly from pre- to post ACZ within groups (not indicated). [file Image_1.tif]

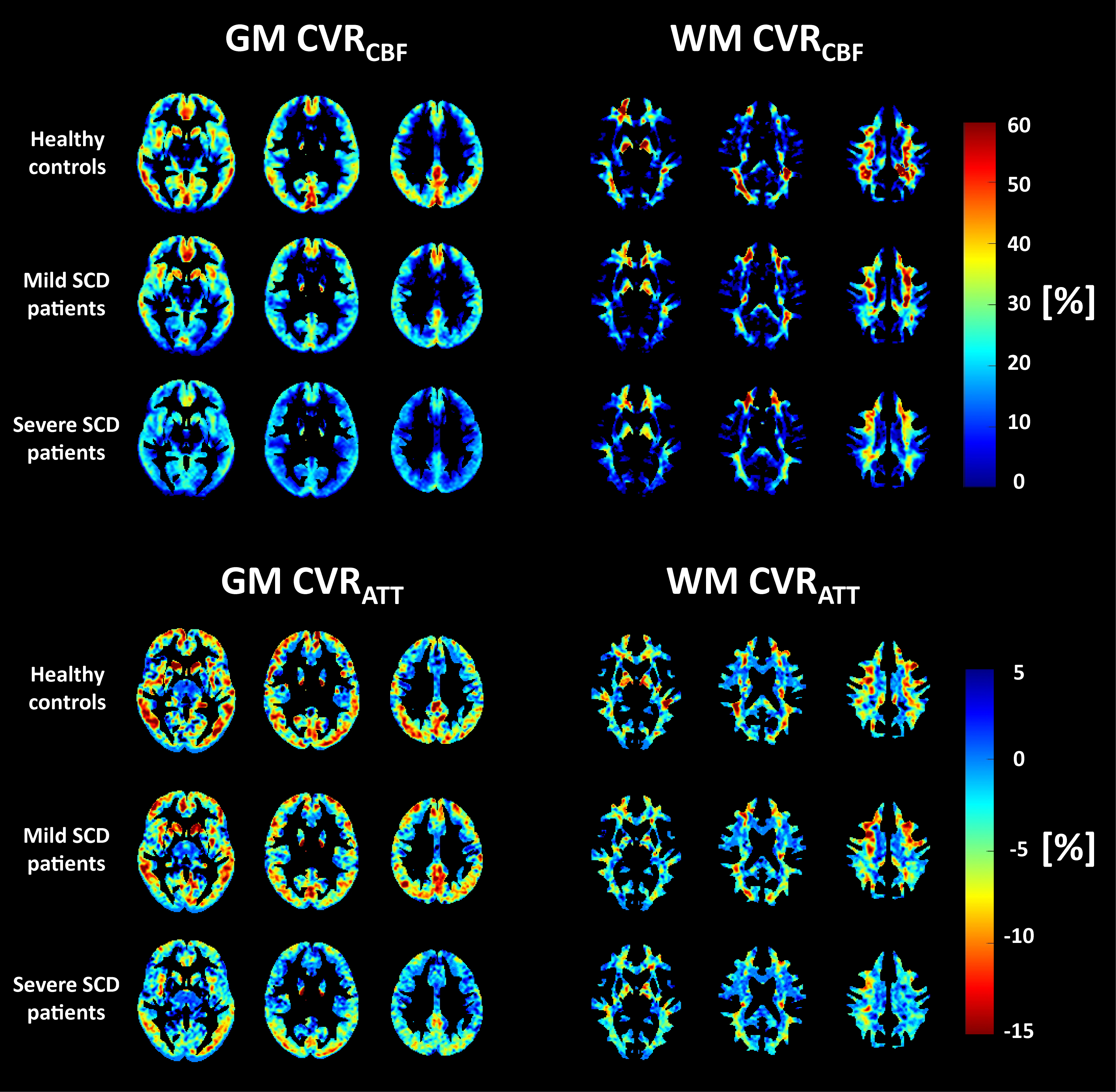

Supplement: Supplementary Figure 2 — Group-averaged CVRCBF and CVRATT maps in GM and WM of healthy controls, mild – and severe patients with SCD. [file Image_2.TIF]

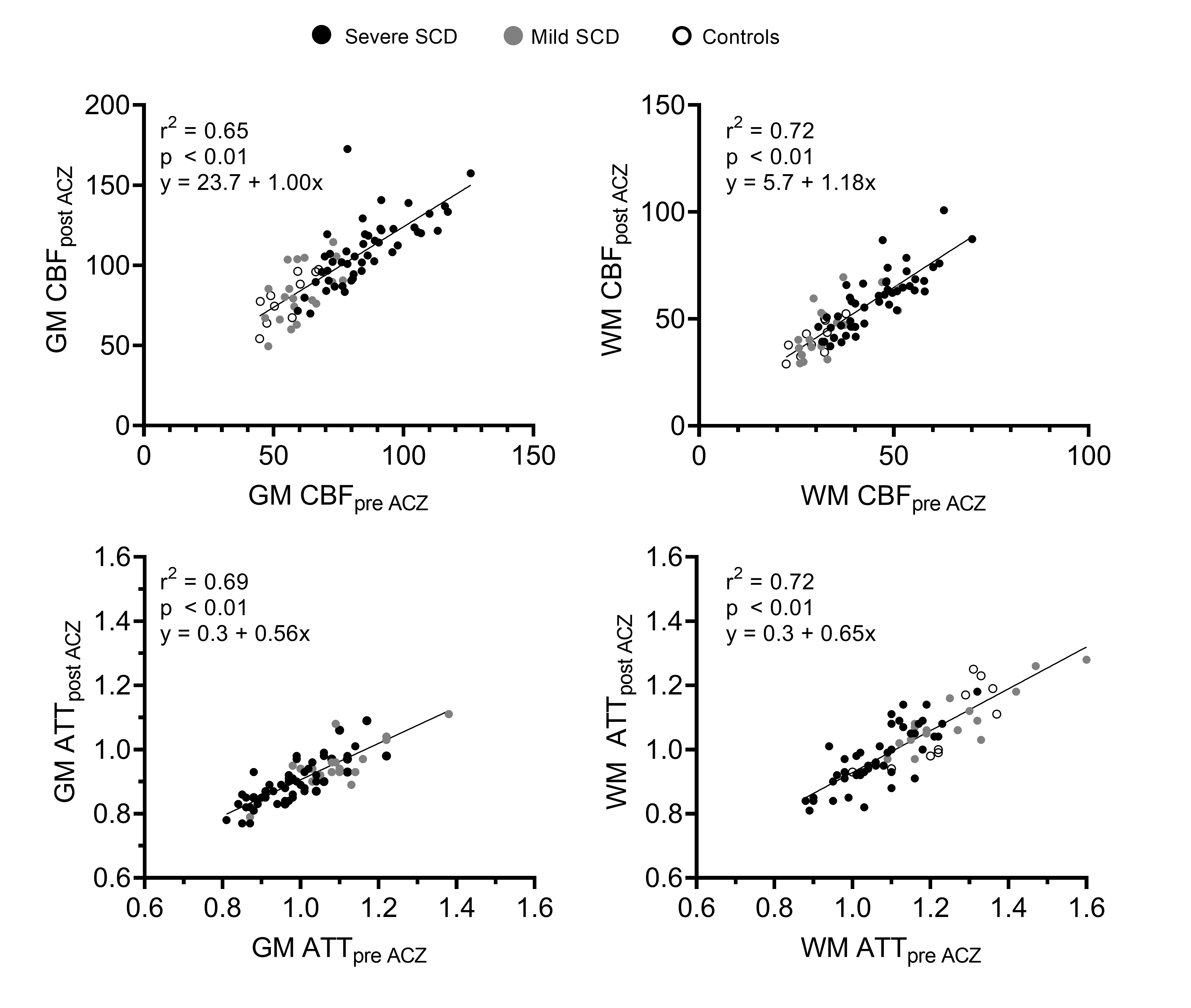

Supplement: Supplementary Figure 3 — Scatterplots displaying correlations between CBFpreACZ and CBFpostACZ and between ATTpreACZ and ATTpostACZ in GM and WM. [file Image_3.tif]
