# Supplementary material for: Impairment of Cerebrovascular Hemodynamics in Patients With Severe and Milder Forms of Sickle Cell Disease
Source: Front Physiol. 2021 Apr 20;12:645205. doi: 10.3389/fphys.2021.645205 (PMC8093944; doi:10.3389/fphys.2021.645205)
Supplement: Supplementary Table 1 — Hb types per subject group. For clarity, patients receiving chronic transfusion therapy were not included. [file Data_Sheet_1.pdf]

Supplementary Material

Supplementary Table 1: Hb types per subject group. For clarity, patients receiving chronic transfusion therapy were not included.

	**Controls**		**Mild SCD patients**		**Severe SCD patients**	
	HbAA	HbAS	HbSC	Hbβ^{+}	HbSS	HBβ^{0}
N	6	4	10	10	44	8
Hb (g/dL)	13.3 ± 1.2	13.4 ± 1.9	12.0 ± 1.1	11.1 ± 1.5	8.7 ± 1.2	8.8 ± 1.5
HbA1 (%)	97.5 ± 0.0	60.3 ± 2.1	-	12.6 ± 3.5	-	-
HbA2 (%)	2.5 ± 0.1	3.4 ± 0.4	4.8 ± 0.5	7.0 ± 0.9	4.6 ± 1.1	5.5 ± 1.2
HbS (%)	-	35.2 ± 2.6	45.4 ± 2.7	72.6 ± 3.8	82.4 ± 14.3	74.2 ± 21.2
HbC (%)	-	-	49.2 ± 2.1	-	-	-
HbF (%)	-	-	2.1 ± 0.1	7.8 ± 6.2	8.7 ± 5.5	11.5 ± 9.2

Supplementary Table 2: demographic information (mean+std) and hemodynamic parameters (median [IQ range]) of patients, divided by treatment group, and test statistics from ANOVA (F) test, Chi square (X^2) and Kruskal-Wallis (H). P-values are shown without multiple comparison correction. Significance level was corrected to 0.017 using Sidak multiple comparison correction. P-values that remain statistically significant after multiple comparison correction are underlined and shown in bold.

	**No prophylactic therapy**	**Hydroxyurea therapy**	**Chronic transfusion therapy**	Test statistics (p-value)	p-value (N-C)*	p-value (N-H)*	p-value (H-C)*
Patient characteristics							
N	33	32	7				
Age (y)	34 ± 12	28 ± 9	21 ± 4	**F: 6.3 (<0.01)**	**<0.01**	0.02	0.09
Sex (Women/Men)	9/24	14/18	5/2	X^2: 5.3 (0.07)	0.04	0.20	0.24
Severe/ Mild SCD	22 / 11	23/9	7 / 0	X^2: 3.2 (0.20)	0.16	0.79	0.17
Blood markers							
Hemoglobin (g/dL)	9.32 ± 2.18	9.81 ± 1.41	8.95 ± 0.74	H: 2.8 (0.25)†	0.76	0.16	0.24
Hematocrit (%)	27.59 ± 6.33	28.17 ± 5.07	26.91 ± 1.90	H: 0.4 (0.81)†	0.75	0.48	0.45
Total Bilirubin (mg/dL)	2.90 ± 2.01	2.83 ± 2.03	1.64 ± 0.77	H: 1.6 (0.44)†	0.24	0.81	0.20
Reticulocytes (10e9/L)	233.61 ± 112.73	200.71 ± 130.73	355.67 ± 153.53	**H: 7.5 (0.02)**†	0.07	0.16	**<0.01**
HbF (%)	4.59 ± 3.05	11.36 ± 6.77	3.38 ± 1.61	**H: 14.1 (<0.01)**†	0.49	**<0.01**	**0.01**
CBF and ATT							
GM CBF pre ACZ (mL/100g/min)	76.5 [31.6]	74.2 [26.4]	81.1 [16.4]	F: 0.3 (0.75)	0.45	0.80	0.54
GM CBF post ACZ (mL/100g/min)	101.8 [34.1]	104.8 [32.0]	104.8 [24.1]	F: 1.3 (0.27)	0.11	0.86	0.15
WM CBF pre ACZ (mL/100g/min)	40.1 [19.6]	38.7 [17.4]	48.4 [13.0]	H: 1.8 (0.41)†	0.18	0.71	0.26
WM CBF post ACZ (mL/100g/min)	54.1 [26.2]	53.9 [23.0]	59.4 [13.8]	H: 0.8 (0.69)†	0.48	0.76	0.39
GM ATT pre ACZ (s)	0.98 [0.15]	0.99 [0.13]	0.99 [0.22]	H: 0.7 (0.69)†	0.78	0.51	0.52
GM ATT post ACZ (s)	0.90 [0.10]	0.90 [0.08]	0.90 [0.15]	H: 0.0 (0.99)†	0.98	0.99	0.97
WM ATT pre ACZ (s)	1.12 [0.18]	1.10 [0.15]	1.11 [0.29]	F: 0.7 (0.52)	0.36	0.68	0.25
WM ATT post ACZ (s)	1.01 [0.15]	1.00 [0.13]	1.00 [0.25]	F: 0.4 (0.69)	0.53	0.69	0.40
**CVR**							
GM CVR_{CBF} (%)	26.9 [31.7]	31.0 [20.6]	35.6 [27.4]	H: 0.0 (0.99)†	0.95	0.94	0.98
WM CVR_{CBF} (%)	31.1 [33.8]	27.8 [24.9]	30.6 [17.1]	H: 1.2 (0.55)†	0.86	0.28	0.66
GM CVR_{ATT} (%)	-4.3 [7.1]	-7.4 [6.6]	-2.8 [9.8]	F: 0.3 (0.73)	0.64	0.62	0.45
WM CVR_{ATT} (%)	-6.6 [8.0]	-7.8 [5.5]	-5.2 [9.9]	F: 0.1 (0.86)	0.60	0.82	0.70

*post-hoc statistics: N-C = patients receiving no prophylactic therapy compared to patients receiving chronic transfusion therapy, N-H = patients receiving no prophylactic therapy compared to patients receiving hydroxyurea therapy, H-C = patients receiving hydroxyurea therapy compared to patients receiving chronic transfusion therapy."†" denote non-parametric tests.

Supplementary Table 3: demographic information (mean+std) and hemodynamic parameters (median [IQ range]) of severe SCD patients only, divided by treatment group, and test statistics from ANOVA (F) test, Chi square (X^2) and Kruskal-Wallis (H). P-values are shown without multiple comparison correction. Significance level was corrected to 0.017 using Sidak multiple comparison correction. P-values that remain statistically significant after multiple comparison correction are underlined and shown in bold.

	**No prophylactic therapy**	**Hydroxyurea therapy**	**Chronic transfusion therapy**	Test statistics (p-value)	p-value (N-C)*	p-value (N-H)*	p-value (H-C)*
**Severe SCD**							
Patient characteristics							
N	22	23	7				
Age (y)	32.3 ± 11.3	27.7 ± 9.6	20.6 ± 4.1	F: 3.9 (**<u>0.03</u>**)	<0.01	0.13	0.10
Sex (Women/Men)	6/16	11/12	5/2	X^2: 4.8 (0.09)	0.07	0.22	0.40
HbSS/ Hbβ0	17/5	20/3	7/0	X^2:2.3 (0.32)	0.30	0.46	0.99
Blood markers							
Hemoglobin (g/dL)	8.06 ± 1.14	9.38 ± 1.2	8.95 ± 0.74	**<u>H: 10.9 (<0.01)</u>**†	0.10	**<u><0.01</u>**	0.48
Hematocrit (%)	24.14 ± 3.91	26.44 ± 4.19	26.91 ± 1.90	H: 4.2 (0.12)†	0.11	0.08	0.74
Total Bilirubin (mg/dL)	3.68 ± 2.17	3.4 ± 2.19	1.64 ± 0.77	H: 4.3 (0.12)†	0.04	0.96	0.05
Reticulocytes (10e9/L)	277.23 ± 112.76	235.93 ± 132.87	355.67 ± 153.53	H: 6.1 (0.05)†	0.45	0.06	0.03
HbF (%)	4.70 ± 3.23	12.20 ± 6.64	3.38 ± 1.61	**<u>H: 13.1 (<0.01)</u>**†	0.49	**<u>0.01</u>**	**<u><0.01</u>**
CBF and ATT							
GM CBF pre ACZ (mL/100g/min)	84.8 [22.9]	81.2 [25.9]	81.1 [16.4]	F: 0.4 (0.66)	0.51	0.41	0.93
GM CBF post ACZ (mL/100g/min)	107.2 [27.7]	107.2 [37.7]	104.8 [24.1]	F: 0.6 (0.55)	0.66	0.42	0.32
WM CBF pre ACZ(mL/100g/min)	44.3 [12.7]	39.2 [18.6]	48.4 [13.0]	F: 0.9 (0.43)	0.88	0.21	0.50
WM CBF post ACZ	60.5 [28.0]	60.0 [23.2]	59.4 [13.8]	F: 1.1 (0.33)	0.55	0.14	0.68
GM ATT pre ACZ (s)	0.94 [0.10]	0.97 [0.11]	0.99 [0.22]	F: 0.8 (0.45)	0.51	0.22	0.88
GM ATT post ACZ (s)	0.88 [0.07]	0.87 [0.07]	0.90 [0.15]	F: 0.4 (0.68)	0.39	0.88	0.45
WM ATT pre ACZ (s)	1.06 [0.16]	1.06 [0.11]	1.11 [0.29]	F: 1.3 (0.27)	0.79	0.11	0.44
WM ATT post ACZ (s)	0.95 [0.13]	0.97 [0.10]	1.00 [0.25]	F: 0.3 (0.72)	0.75	0.42	0.82
**CVR**							
GM CVR_{CBF} (%)	26.0 [27.7]	30.6 [16.0]	35.6 [27.4]	H: 0.3 (0.87)†	0.80	0.72	0.63
WM CVR_{CBF} (%)	27.9 [34.6]	27.8 [24.9]	30.6 [17.1]	H: 0.6 (0.76)†	0.93	0.50	0.59
GM CVR_{ATT} (%)	-3.3 [3.8]	-5.4 [7.8]	-2.8 [9.8]	H: 1.4 (0.50)†	0.78	0.23	0.61
WM CVR_{ATT} (%)	-5.3 [5.6]	-7.6 [6.8]	-5.2 [9.9]	F: 0.1 (0.93)	0.89	0.70	0.90

*post-hoc statistics: N-C = patients receiving no prophylactic therapy compared to patients receiving chronic transfusion therapy, N-H = patients receiving no prophylactic therapy compared to patients receiving hydroxyurea therapy, H-C = patients receiving hydroxyurea therapy compared to patients receiving chronic transfusion therapy."†" denote non-parametric tests.

Supplementary Table 4: demographic information (mean+std) and hemodynamic parameters (median [IQ range]) of mild SCD patients only, divided by treatment group. Statistically significant p values are underlined and shown in bold.

	**No prophylactic therapy**	**Hydroxyurea therapy**	**Chronic transfusion therapy**	p-value
**Mild SCD**				
Patient characteristics				
N	11	9	0	
Age (y)	37.6 ± 12.4	28.4 ± 8.6	-	0.07
Sex (Women/Men)	3/8	3/6	-	0.99
HbSC/ Hbβ+	7/4	3/6	-	0.37
Blood markers				
Hemoglobin (g/dL)	11.84 ± 1.40	10.87 ± 1.54	-	0.25†
Hematocrit (%)	34.50 ± 4.14	32.50 ± 4.62	-	0.66†
Total Bilirubin (mg/dL)	1.59 ± 0.43	1.76 ± 1.18	-	0.91†
Reticulocytes (10e9/L)	146.36 ± 37.67	108.26 ± 66.50	-	0.18†
HbF (%)	4.11 ±2.43	8.85 ± 7.07	-	0.26†
CBF and ATT				
GM CBF pre ACZ (mL/100g/min)	56.0 [11.1]	64.2 [13.9]	-	**0.03**
GM CBF post ACZ	79.3 [19.1]	96.6 [28.3]	-	0.09
WM CBF pre ACZ(mL/100g/min)	28.4 [9.5]	33.4 [18.6]	-	**0.02**†
WM CBF post ACZ	40.2 [15.1]	52.3 [22.8]	-	0.21†
GM ATT pre ACZ (s)	1.09 [0.10]	1.06 [0.12]	-	0.86
GM ATT post ACZ (s)	0.94 [0.10]	0.94 [0.04]	-	0.64
WM ATT pre ACZ (s)	1.25 [0.17]	1.17 [0.10]	-	0.40
WM ATT post ACZ (s)	1.06 [0.12]	1.07 [0.04]	-	0.74
**CVR**				
GM CVR_{CBF} (%)	40.1 [46.4]	37.4 [45.1]	-	0.62
WM CVR_{CBF} (%)	44.6 [33.0]	26.1 [41.9]	-	0.43
GM CVR_{ATT} (%)	-8.24 [9.61]	-9.98 [8.74]	-	0.80
WM CVR_{ATT} (%)	-11.14 [8.40]	-8.24 [8.43]	-	0.34

* "†" denote non-parametric tests.

Regarding supplementary Table 4: in patients with mild SCD, GM and WM CBF at baseline was significantly higher in patients receiving hydroxyurea compared to patients receiving no prophylactic therapy. We are not sure what causes this difference in baseline CBF among mild SCD patients. A possible explanation could be the lower hemoglobin and higher HbF. We have shown that both these effects increase baseline CBF. Furthermore, the differences in number of

patients with HbSC and Hbβ+ between the group with or without hydroxyurea therapy could introduce unexpected differences. Additionally, hydroxyurea treatment is dependent on clinical presentation and patients' preferences which could cause a bias in the analysis of the effect of hydroxyurea on hemodynamics of the cerebral perfusion. Therefore, we believe these results should be interpreted with caution.

Supplementary analysis on ΔCBF and ΔATT

To get a closer understanding which factors affect the increase in CBF and reduction in ATT after ACZ administration, an alternative analysis was performed on the difference between pre ACZ and post ACZ CBF and ATT values. First, ΔCBF, defined as CBF post ACZ – CBF pre ACZ, and ΔATT, defined as ATT post ACZ – ATT pre ACZ, were calculated for GM and WM. Next, stepwise multiple regression was performed between the resulting difference values and the following predictors: the corresponding baseline condition, age, sex, participant groups, hemoglobin, total bilirubin, reticulocytes and fetal hemoglobin (HbF), using pairwise deletion to account for missing values. Variables with $p<0.05$ were retained in the multiple regression model as a significant predictor for ΔCBF or ΔATT.

For ΔCBF in GM and WM, only sex was a statistically significant predictor (GM: $r^2 = 0.16$; $p<0.01$ and WM; $r^2 = 0.16$, $p<0.01$). For ΔATT in GM, only the corresponding baseline conditions was a statistically significant predictor ($r^2 = 0.58$; $p<0.01$). For WM ΔATT, baseline ATT and sex were statistically significant predictors (baseline ATT; $\beta = -0.70$, $p<0.01$ and sex; $\beta = -0.26$, $p = 0.026$).

For GM CBF and ATT, both models result in similar results. In contrast to the model in the main manuscript, HbF was not a significant predictor for WM ΔCBF. In this analysis, neither 1/Hb nor HbF was statistically significant when both added to the model. (HbF; $\beta = -0.22$, $p = 0.14$, 1/Hb; $\beta = 0.34$, $p = 0.17$). When considered separately, both were significant. (Sex; $\beta = 0.34$, $p<0.01$ and HbF; $\beta = -0.30$, $p = 0.03$ or Sex; $\beta = 0.36$, $p<0.01$ and 1/Hb; $\beta = 0.48$, $p = 0.02$). This is slightly different than the result from the model in the main manuscript, where sex and HbF were both significant predictors of WM CBF after correcting for baseline conditions.

Additionally, this analysis shows that in GM and WM, baseline conditions were significant predictors for ΔATT but not for ΔCBF. Thus, ΔCBF is independent of baseline CBF but ΔATT is not independent of baseline ATT. This can also be observed from supplementary Figure 3 where the slopes of the linear fit between pre ACZ CBF and post ACZ CBF are close to one (GM: 1.00 and WM: 1.18) but the slopes of the linear fit between pre ACZ ATT and post ACZ ATT are not (GM: 0.56 and WM: 0.65).
